# Effect of Low-power Laser on Treatment of Orofacial Pain

**DOI:** 10.5681/joddd.2010.019

**Published:** 2010-09-16

**Authors:** Hamid Reza Khalighi, Fahimeh Anbari, Jamileh Beygom Taheri, Sedigheh Bakhtiari, Zahra Namazi, Firoz Pouralibaba

**Affiliations:** ^1^ Assistant Professor, Department of Oral Medicine, Faculty of Dentistry, Shahid Beheshti University of Medical Sciences, Tehran, Iran; ^2^ Postgraduate Student, Department of Oral Medicine, Faculty of Dentistry, Shahid Beheshti University of Medical Sciences, Tehran, Iran; ^3^ Associate Professor, Department of Oral Medicine, Faculty of Dentistry, Shahid Beheshti University of Medical Sciences, Tehran, Iran; ^4^ Assistant Professor, Department of Oral Medicine, Faculty of Dentistry, Shahid Beheshti University of Medical Sciences, Tehran, Iran; ^5^ Dentist, Private Practice, Tehran, Iran; ^6^ Assistant Professor, Department of Oral Medicine, Faculty of Dentistry, Tabriz University of Medical Sciences, Tabriz, Iran

**Keywords:** Laser, low-power laser, orofacial pain

## Abstract

Low-power lasers are a group of lasers with a power less than 250 mW and unlike high-power lasers they have no effect on tissue temperature; they produce light-dependent chemical reactions in tissues. These lasers have analgesic features with their ability to trigger reactions that reduce pain and inflammatory mediators. Low-power lasers can also be used instead of needles in acupuncture to decrease pain. Due to these features they have been used in the treatment of orofacial pain, including tooth hypersensitivity, post-operative flare-ups, mucositis, facial myalgia, temporomandibular joint disorders and neuralgia. In this article we review the effects of low-power lasers and their success rate in different studies. As the name implies (LASER: Light Amplification by the Stimulated Emission of Radiation), laser amplifies light by stimulated and excited radiation; in other words, it is amplification of excited light emission. Such radiation usually has some characteristic features, including mono-chromaticity, coherency, high intensity and polarity. There are various classifications for lasers based on their active material (solid, fluid and gas), wavelength, emission type and power.

## Introduction

Based on power, lasers can be classified into the following three categories:

*I. High-power lasers (hard, hot)*

These lasers increase tissue kinetic energy and produce heat. As a result, they leave their therapeutic effects through thermal interactions. These effects include necrosis, carbonization, vaporization, coagulation and denaturation. These lasers usually have an output power of more than 500 mW.^1 , 2^

*II. Intermediate-power lasers*

These lasers leave their therapeutic effects without producing significant heat. To shorten treatment period length and to accelerate the therapeutic effect in some cases, low-power lasers are replaced by intermediate lasers with output powers ranging from 250-500 mW.^1 , 2^

*III. Low-power lasers (soft, cold)*

These lasers have no thermal effect on tissues and produce a reaction in cells through light, called photobiostimulation or photobiochemical reaction. Output power of these lasers is less than 250 mW.

The critical point that differentiates low-power lasers from high-power ones is photochemical reactions with or without heat. The most important factor to achieve this feature in lasers is not their power but the power density per cm^2^. If the density is lower than 670 mW/cm^2^,it can mimic stimulatory effect of low-power lasers without any thermal effects.^1 , 2^

### Analgesic effects of laser

Stimulation of any point of the body creates neural impulses that are transmitted to upper nervous centers by neurons that have different features. These impulses finally reach the CNS.

Low-power lasers can leave their effects in different parts of the body. Currently the following analgesic effects are recognized:

1. Low-power lasers inhibit the release of mediators from injured tissues. In other words, they decrease concentration of chemical agents such as histamine, acetylcholine, serotonin, H^+^ and K^+^ ,all of which are pain mediators.
2. Low-power lasers inhibit concentration of acetylcholine, a pain mediator, through increased acetylcholine esterase activity.
3. They cause vasodilatation and increase blood flow to tissues, accelerating excretion of secreted factors. On the other hand, better circulation leads to a decrease in tissue swelling.
4. They decrease tissue edema by increasing lymph drainage. They also remove the pressure on nerve endings, resulting in stimulation decrease.
5. These lasers decrease sensitivity of pain receptors as well as transmission of impulses.
6. They decrease cell membrane permeability for Na ^+^ and K ^+^ and cause neuronal hyperpolarization, resulting in increased pain threshold.
7. Injured tissue metabolism is increased by electromagnetic energy of laser. This is induced by ATP production and cell membrane repolarization.
8. Low-power lasers increase descending analgesic impulses at dorsal spinal horn and inhibit pain feeling at cortex level.
9. They balance the activity of adrenalin and noradrenalin system (autonomous system) as a response to pain.
10. Low-power lasers increase the urinary excretion of serotonin and glucocorticoids, increasing the production of β-endorphin.

### Reflexotherapy or laser acupuncture

At present acupuncture is generally accepted as an adjunctive treatment, with documented analgesic effects on different kinds of pain. In this method specific points of the body are selected and stimulated with needles that are inserted into various depths, which resultant analgesia. Low-power lasers can be used for stimulation instead of needles. Access to different depths is possible by applying low-level lasers with different wavelengths and changing the output power. This can have the same effect as acupuncture. Furthermore, there will be no pain, discomfort, inflammation and cross-contamination compared to needle use.^3^

###  Effect of low-level laser on maxillofacial pain

Maxillofacial pain has different origins such as teeth, mucosa, muscles, nerves and vessels. Since most of these tissues are within reach, low-level lasers can be used to initiate most of its previously mentioned effects.

### 1. Effect of low-level laser on toothache

*A. Toothache of dentinal origin*

In addition to caries, other lesions such as erosion, abrasion, inappropriate restorations and gingival recession, which expose the root, may induce toothache of dentinal origin. There are different ways to reduce dentin hypersensitivity, including fluoridated varnish, meticulous hygiene, desensitizing agents, restoration of exposed areas with restorative materials and covering the tooth with crowns.^4 , 5^

Brugnera et al^6^ used He-Ne low-power laser to treat 300 patients with dentin hypersensitivity in 1995-1997. The success rate was reported to be 92%. Compared to the control group there was a significant difference between patients’ complaints after application of low-level laser on apical and cervical segments of teeth for one minute and this difference was greater after the second and third laser applications.^7^ Corona et al^8^ showed that Ga-Al-As low-level laser has the same effect as fluoridated varnish.

*B. Effect of low-level laser on preventing or eliminating pain after surgical removal of third molars*

Although studies in 1990s indicated that low-level lasers have no effect on pain after third molar surgery,^9 , 10^ Marković & Todorović^11^ showed that patients who received 100 mg of diclofenac sodium before surgery and were also exposed to laser after surgery had less pain compared to those who only received 100 mg of diclofenac sodium.

Bjordal et al^12^studied the effect of different doses of low-power laser on pain after third molar surgery in 658 patients and concluded that 0.37-0.96 J ⁄cm^2^laser had no effect on eliminating symptoms but 6-7 J laser reduced pain to a greater degree. Therefore, there is a need for more research on low-level lasers in the treatment of pain to reach the optimal dose.

*C. Effect of low-level laser on post-operative pain in endodontics*

Previous studies have shown that exposure of the gingiva over periapical area to low-level laser with 809-nm wavelength can reduce post-operative endodontic pain compared to control groups. However, differences in the severity of pain between the two groups a few days after treatment is more noticeable.^13^

*D. Effect of low-level laser on reducing post-orthodontic pain*

Earlier studies have not reported any significant differences between the patients who received laser after placement of brackets and those who were exposed to placebo,^14^ but Turhani et al^15^ reported that exposure to 670-nm wavelength laser resulted in significant pain relief during the first 6 hours after placement of brackets compared to the control group. This trend remained the same for 30 hours after treatment, but there were no significant differences between the two groups after 54 hours.

### 2. Effect of low-level laser on mucositis pain

Maiya & Fernande^16^ showed that in patients who had oral mucositis because of radiotherapy of neck and head region, exposure to 632.6-nm wavelength decreased pain more than that in those who received oral analgesics or topical anesthesia. Mucositis pain following chemotherapy can also be reduced by low-level laser with a wavelength of 650 nm.^17^ In addition, it has been shown that low-level lasers have prophylactic effect on mucositis following chemotherapy.^18 , 19^

###  3. Effect of low-level laser on myofacial pain

Several studies have shown that use of 830-nm wavelength laser in several appointments can reduce or eliminate myofacial pain.^20 , 21^ Altofini et al^22^reported no pain in their patients up to 3 months. Furthermore, effectiveness of laser acupuncture has been confirmed in decreasing myofacial pain.^23^

### 4. Effect of low-level laser on temporomandibular joint disorder pain

Kulokciglu et al^24^ showed decrease in pain related to temporomandibular joint disorders in 35 patients. In another study pain decreased significantly in patients suffering from temporomandibular joint disorders, and exposed to 785-nm laser compared to the placebo group. They also had no pain during the 6-month follow-up period.^25^

### 5. Effect of Low-level laser on trigeminal neuralgic pain

According to Eckerdal & Bastin^26^ low-level laser of 830-nm wavelength was efficient in the treatment of 81% of patients, with 42% of them having no pain after a year. In contrast, there was an improvement in 50% of patients who had been treated with injection of alcohol and only 20% remained pain-free after a year. It has also been shown that compared to placebo, low-level laser is significantly effective in pain relief.^27^ The effectiveness of low-level laser in the prevention and treatment of post-herpetic neuralgia has also been confirmed in several studies.^28 , 29^

## Conclusion

As mentioned before, low-level lasers cause photobiochemical reactions that result in pain relief. Considering the effect of neurotransmitters on nerves, lasers are expected to be effective in eliminating all kinds of pain that result from nerve irritation and nociceptor excitation (neuropathic pain). If location of inflammation is within reach, lasers can reduce pain of inflammatory origin through their anti-inflammatory properties. If irritated and inflamed sites are not accessible, laser acupuncture can be used. Although low-level lasers have been shown to be effective in improving oral and maxillofacial pain, they are not used widely. The need for several appointments and the novelty of the procedure limit the widespread use of lasers.
